# Supplementary material for: WordCommentsAnalyzer: A windows software tool for qualitative research
Source: F1000Res. 2018 Sep 4;7:536. Originally published 2018 May 3. [Version 2] doi: 10.12688/f1000research.14819.2 (PMC6137411; doi:10.12688/f1000research.14819.2)
Supplement: Supplementary file 3 [file f1000research-7-17614-s0002.tgz › b2a79496-4ada-47c4-9682-a99ce867836d.zip!/Eight Iranian Health Journals/Yafteh _26.docx]

© Yafteh  . This work is licensed under CC BY 4.0 (https://creativecommons.org/licenses/by/4.0/).

Volume & Issue: دوره 14 - شماره 2

Title: ویژگی های آنتی اکسیدانی عصاره هیدروالکلی و اسانس برگ گیاه ویتکس (Vitex pseudo-negundo)

http://yafte.lums.ac.ir/article-1-748-fa.html

مقدمه: آنتی اکسیدان ها باعث محافظت بدن در برابر تنش اکسیداتیو ناشی از رادیکال های آزاد می شوند. هدف از این مطالعه مقایسه ویژگی های آنتی اکسیدانی عصاره هیدروالکلی و اسانس برگ گیاه ویتکس است. مواد و روش‌ها:در این مطالعه عصاره هیدروالکلی و اسانس برگ گیاه ویتکس تهیه شد وویژگی های آنتی اکسیدانی نمونه ها با استفاده از روش های مختلف مورد ارزیابی قرار گرفت. توان حذف رادیکال های آزاد از طریق روش 2و 2 دی فنیل - 1- پیکریل هیدرازیل (DPPH) اندازه گیری شد. ظرفیت تام آنتی اکسیدانی با استفاده از روش فسفومولیبدات اندازه گیری شد. همچنین میزان ترکیبات فنلی وفلاونوئیدی نمونه ها با استفاده از روش فولین سیوکالتو و زیشن بدست آمد. یافته‌ها: نتایج به دست آمده نشان داد که ظرفیت تام آنتی اکسیدانی عصاره هیدروالکلی و اسانس برگ ویتکس به ترتیب برابر با (25/0±76/1 60/0±12/2)، میزان فنل عصاره هیدروالکلی و اسانس برگ ویتکس به ترتیب برابر با (2±22 3±11/133)، میزان فلاوونویید عصاره هیدروالکلی و اسانس برگ ویتکس به ترتیب برابر با (1±18 4±5/5) است. میزان IC₅₀ عصاره هیدروالکلی، اسانس و بوتیلیتد هیدروکسی تولوئن (BHT) به عنوان کنترل مثبت به ترتیب برابر با (52/4±75/224 3± 00/133 1±88/3 میکروگرم برمیلی لیتر) است. بحث و نتیجه‌گیری: نتایج بدست آمده نشان داد که ویتکس منبع خوب و قابل دسترسی از ترکیبات آنتی اکسیدانی است و می‌توان از آن در صورت مفید بودن در فراورده‌های غذایی، دارویی و صنعتی استفاده کرد.

Title: خصوصیات فیزیکی ‌و ارزش تغذیه‌ای ارقام زرد و قرمز سیب لبنانی (Malus, domestica Borkh) تولیدی لرستان

http://yafte.lums.ac.ir/article-1-749-fa.html

مقدمه: سیب میوه‌ای است که دارای ارزش تغذیه‌ای و درمانی است. هدف از این مطالعه تعیین میزان برخی از ترکیبات مغذی سیب قرمز و زرد لبنانی تولید شده در لرستان بود. مواد و روش‌ها: نمونه‌های مورد نیاز از باغات استان در اواخر شهریورماه سال 1387 جمع‌آوری شد. نمونه ها به مدت 2 روز در دمای 80 درجه سانتیگراد خشک شد. نمونه های خشک شده جهت آنالیز مواد مغذی آسیاب گردید. فسفر با روش اسپکتروفتومتری ، کلسیم، آهن و روی با دستگاه جذب اتمی، سدیم و پتاسیم با فلیم فتومتر اندازه‌گیری گردید. سایر فاکتورهای اندازه‌گیری شده شامل انرژی، آب، پروتئین، فیبر، خاکستر و کربوهیدرات‌ها بود. بحث و نتیجه‌گیری: ارزش تغذیه ای سیب استان از نظر مقدار انرژی، کربوهیدرات و کلسیم بیشتر از مقادیر موجود در جدول ترکیبات مواد غذایی ایران و از نظر مقدار آهن کمتراز آهن گزارش شده در این جدول است. یافته‌ها: میزان آب، مواد جامد محلول، خاکستر و فیبر در سیب قرمز به ترتیب 40/2، 78، 70/1، و 9/17 درصد و در سیب زرد به ترتیب 42/2، 98/77، 72/1، و 88/17 درصد بود. مقدار انرژی، قند و پروتئین سیب قرمز به ترتیب 73 کالری، 18 و 27/0 گرم در 100 گرم و در سیب زرد به ترتیب 83 کالری، 6/20 و 2/0 گرم بود.مقدار پتاسیم، کلسیم، فسفر، سدیم، آهن و روی در سیب قرمز به ترتیب8/63، 6/27، 9، 8/4، 24/0 و 14/0 میلی‌گرم و در سیب زرد به ترتیب 3/66،7/27 ،8/8، 5/4، 23/0و 14/0 میلی‌گرم در 100 گرم بود.

Title: بررسی اثر گیاه مرزه خوزستانی(Satureja khuzestanica) بر تشنج ناشی ازنیکوتین در موش سوری

http://yafte.lums.ac.ir/article-1-750-fa.html

مقدمه: تشنج از مهمترین علائم صرع و بسیاری از اختلالات نورولوژیکی است . با توجه به عوارض جانبی داروهای صناعی و عدم پاسخ‌دهی مناسب به درمان‌های موجود، امروزه داروهای گیاهی بطور وسیعی در کنترل تشنج به کار می‌روند. گیاه مرزه خوزستانی((Satureja khuzestanica یک گیاه بومی ایران است که اثرات ضد دردی و ضد اسپاسمی دارد. لذا با توجه به این اثرات و اترات مهاری آن بر کانال‌های کلسیمی در این مطالعه اثرات اسانس روغنی این گیاه بر تشنج ناشی از نیکوتین مورد بررسی قرار گرفت. بحث و نتیجه‌گیری: نتایج فوق بیانگر این واقعیت است که اسانس گیاه مرزه دارای اثر ضد تشنج می باشد. شناخت مکانیسم‌های دقیق آن نیازمندبه مطالعات وسیع‌تری است. یافته‌ها: نتایج حاصل نشان داد که اسانس مرزه وکارواکرول در دوز80 میلی‌گرم بر کیلوگرم موجب افزایش زمان شروع تشنج و کاهش شدت و دوام تشنج می‌شود. مواد و روش‌ها: در این مطالعه تجربی از 40 موش سفید کوچک نر استفاده گردید. حیوانات به 5 گروه 8 تایی تقسیم شدند. اسانس روغنی مرزه به مقادیر40،80،120 میلی‌گرم بر کیلوگرم وکارواکرول با دوز80 میلی‌گرم بر کیلوگرم به صورت داخل صفاقی تزریق شد. گروه شاهد توئین20 (3%) هم حجم اسانس دریافت نمودند. بعد از 30 دقیقه، به تمام گروه‌ها نیکوتین (5 میلی‌گرم بر کیلوگرم) از طریق داخل صفاقی تزریق گردید و زمان شروع، شدت و دوام تشنج مورد بررسی قرار گرفت.

Title: خالص سازی و بررسی خواص سینتیکی آنزیم هیستامیناز از گونه خلر ( Lathyrus sativus ) با استفاده از کروماتوگرافی تعویض آنیون

http://yafte.lums.ac.ir/article-1-751-fa.html

مقدمه: مسمومیت با هیستامین به طور مستقیم در ارتباط با نگهداری نامناسب غذاها می‌باشد. دی آمین اکسیداز، دآمیناسیون اکسیداتیو هیستامین و آمین‌های بیوژنیک دیگر مانند کاداورین و پوترسین را کاتالیز می‌کند. دی‌آمین اکسیدازهای گیاهی پراکندگی زیادی در خانواده لگومینه مانند نخود، خلر و ماش دارند. به این دلیل، این مطالعه انجام گرفت تا خالص‌سازی و خواص کینتیکی آنزیم هیستامیناز از جوانه‌های خلر آزمایش گردد. بحث و نتیجه‌گیری: در مجموع آنزیم هیستامیناز گیاهی به عنوان یک آنزیم جدید در پیشگیری از مسمومیت با هیستامین امیدوار کننده است یافته‌ها: فعالیت ویژه آنزیم نهایی خالص شده برای سوبسترای هیستامین 50 واحد در میلی‌گرم پروتئین یادداشت شد و خواص کینتیکی آنزیم (05/3=Vmax ، 5/2=Km) و هموژنوسیتی آنزیم خالص شده بوسیله SDS-PAGE در ناحیه 72 کیلو دالتون نشان داده شد. مواد و روش‌ها: خالص‌سازی آنزیم بوسیله روش انجام شده فدریکو و همکاران با یکسری تغییرات بود. به طور کوتاه، با استفاده از رسوب دادن بوسیله سولفات آمونیوم، دیالیز علیه بافر پتاسیم 50 میلی مولار 7pH= به مدت 18 ساعت، تغلیظ بوسیله کیسه‌های دیالیز بر روی پلی اتیلن گلیکول، گذاشتن روی ستون DEAE-52 سفادکس که قبلاً با بافر فسفات پتاسیم 15 میلی مولار و 5/5pH= به تعادل رسیده بود حاوی یک میکرومول CuCl2 و در نهایت آنزیم با افزایش شیب نمک در M 2/0 خارج گردید. هموژنوسیته آنزیم بوسیله تکنیک SDS-PAGE در هر مرحله تائید شد و فعالیت دی‌آمین اکسیداز بوسیله اسپکتروفتومتر مشخص گردید.

Title: بررسی تأثیرعصاره هیدروالکلی سرخارگل بر پارامترهای خون در موش کوچک آزمایشگاهی

http://yafte.lums.ac.ir/article-1-752-fa.html

مقدمه: سرخارگل با نام علمی Echinacea purpurea متعلق به خانواده گل ستاره‌ای‌ها (Asteraceae) دارای اثرات درمانی زیادی است که یکی از مهم‌ترین آنها تقویت سیستم ایمنی می باشد در این مطالعه اثرعصاره هیدروالکلی سرخارگل بر پارامترهای خونی موش‌های کوچک آزمایشگاهی (گونهBalb/C) بررسی شده است. مواد و روش‌ها: در این تحقیق 48 موش در 6 گروه به طور تصادفی تقسیم شدند.گروه کنترل هیچ عصاره‌ای دریافت نکرد.گروه دارونما cc (placebo) 5/ 0 نرمال سالین به صورت یک روز در میان و 4 گروه تیماری به ترتیب عصاره با دوزهای ) mg/kg/2day50،100،200،300 ) به صورت تزریق درون صفاقی(IP) دریافت کردند. پس از آن از همه موش‌ها به روش خون‌گیری از قلب، نمونه خون گرفته شد. نتایج بدست آمده با نرم‌افزار آماری SPSS در سطح 05/0P< آنالیز گردید. بحث و نتیجه‌گیری: یافته‌های این مطالعه نشان می‌دهد عصاره سرخارگل احتمالاً می‌تواند با افزایش در تعداد سلول‌های سفید خون باعث تقویت سیستم ایمنی شده و با افزایش در تعداد سلول‌های قرمز خون بر خون‌سازی موثر باشد. یافته‌ها: مطالعه پارامترهای خونی نشان دادکه تعداد کل سلول های سفید خون هر 4 گروه تجربی در مقایسه با گروه کنترل افزایش معنی‌دار دارد(P<0.05). تعداد نوتروفیل‌ها در هر 4 گروه تجربی در مقایسه با گروه کنترل کاهش معنی‌دار نشان داده است، اما تفاوت معنی‌داری در مونوسیت‌ها مشاهده نشد. تعداد سلول‌های قرمز خون در گروه‌های 1 و 2 در مقایسه با گروه کنترل افزایش معنی‌دار دارد. در این مطالعه عصاره بر میانگین هموگلوبین، هماتوکریت و شاخص‌های سلول‌های قرمز خون(MCHC، MCH ،MCV هیچ اثر معنی‌داری را از نظر آماری نشان نمی‌دهد.

Title: بررسی تاثیر مراحل فنولوژیک بر روی درصد و ترکیبات شیمیایی اسانس گیاه مرتعی Satureja hortensis L.

http://yafte.lums.ac.ir/article-1-753-fa.html

مقدمه: Satureja hortensis L گیاهی است علفی ، یک‌ساله دارای ساقه ای منشعب به طول 10 تا 30 سانتیمتر که به دلیل دارا بودن ظاهری به رنگ سبز متمایل به خاکستری ، ازگونه های مشابه قابل تشخیص می باشدو برگ‌های آن باریک و بلند است. در سال های اخیر خواص ضد ویروسی، ضد التهاب، ضد باکتری، ضد قارچی، ضد اسپاسم، تقویت کننده معده و تسهیل کننده هضم این گونه به اثبات رسیده است. مواد و روش‌ها: این تحقیق با هدف بررسی نوع و مقدار ترکیبات اسانس گیاه مرزه تابستانه در دو مرحله فنولوژیک رویشی و زایشی انجام گرفت. جهت شناسایی میزان درصد ترکیبات اسانس گیاه تعداد 30 نمونه به طور تصادفی از ارتفاعات شاطر آباد در دو مرحله فنولوژیک رویشی و زایشی گیاه برداشت شد و اسانس آن توسط دستگاه کلونجر تهیه شد. جهت شناسایی ترکیبات تشکیل دهنده اسانس از دستگاه GC/MSاستفاده شد در تمامی طیف های داده شده GC/MS از روی الگوی خروج آلکانهای نرمال و شاخص بازداری طیف ها ، اندیس کوواتس برای تک تک پیک ها محاسبه گردید. بحث و نتیجه‌گیری: نتایج نشان می دهد که از نظر نوع ترکیبات اصلی اسانس گیاه مرزه تابستانه در دو مرحله رویشی و زایشی تفاوت قابل توجهی ندارد، اگرچه از نظر تعداد ترکیبات شناسایی شده تغییرات نسبتا زیادی در دو مرحله فنولوژیک ذکر شده دیده می شود. همچنین نتایج بدست آمده از این تحقیق نشان داد که سه ترکیب اصلی اسانس گیاه مرزه تابستانه به ترتیب تیمول، گاما ترپینن، و کارواکرول می باشد که خواص ضد باکتریایی، ضد قارچی و آنتی اکسیدانی آنها، خاصیت دارویی و اثرهای درمانی اسانس این گیاه را بطور کامل تایید می کند. یافته‌ها: میزان اسانس کل گیاه در دو مرحله فنولوژیک قبل از گل دهی(مرحله رویشی) و بعد از گل دهی(مرحله زایشی) به ترتیب 5/0 و 4/0 در صد(وزنی-حجمی) بود. تعداد ترکیبات شناسایی شده در مرحله رویشی 27 ترکیب و برای مرحله زایشی 19 ترکیب بود که بر اساس آن نزدیک به 98% اسانس گیاه شناسایی گردید.

Title: مقایسه کمی و کیفی اسانس اکوتیپ‌های مختلف مریم نخودی (Teucrium polium L. ) در رویشگاه های مختلف استان لرستان

http://yafte.lums.ac.ir/article-1-754-fa.html

مقدمه: مریم نخودی گیاهی است از خانواده Lamiaceae متعلق به جنس Teucrium علفی، پایا، کرکدار و معطر با ارتفاع 40-10 سانتی‌متر، این گیاه حاوی مقادیری تانن، ترپنوئید، ساپونین، استرول، فلاونویید و لوکوآنتوسیانین است همچنین واجد اثرات آنتی‌باکتریال بوده ولی اثرات ضد قارچی‌بارزی ندارد . از خواص دارویی این گیاه می توان به اثرات کاهنده کلسترول و تری گلیسیرید و پائین آورنده فشارخون، آنتی‌اکسیدان، ضدتب و ضدمیکروب، ضددرد، آنتی اولسری، و آنتی اسپاسمودیک اشاره داشت. مواد و روش‌ها: در این پژوهش از اکوتیپ های مختلف مریم نخودی سرشاخه های گلدار بصورت نمونه گیری سیستماتیک تصادفی تهیه گردید و به روش تقطیر با آب بوسیله دستگاه کلونجر اسانس آن استخراج شد سپس توسط دستگاه های GC و GC/MS ترکیبات شیمیایی آن شناسایی گردید.داده های بدست آمده توسط نرم افزار آماری Spss ver 19 مورد آنالیز قرار گرفت. بحث و نتیجه‌گیری: نتایج بررسی انجام شده نشان داد که 4 گونه از جنس Teucrium در استان لرستان رویش دارد که عبارتنداز:Teucrium orientale ، Teucrium polium، Teucrium melissoides ، Teucrium stocksianum پس از بررسی نتایج بدست آمده مشخص گردید که اکوتیپ خرم آباد از نظر عملکرد اسانس ( 6/0 درصد ) همچنین تعداد ترکیبات شیمیایی شناسایی شده موجود در اسانس از سایر اکوتیپ ها برتر می باشد .پس از آنالیز داده های مربوط به ترکیبات شیمیایی شناسایی شده اکوتیپ های مورد بررسی مریم نخودی در استان لرستان مشخص شد چنانچه اولویت تولید ترکیبات شیمیایی نظیر : cavacrol،bicyclogermacrene ، β-pinene، β-caryophyllene، farnesene-cis-bباشد اکوتیپ خرم آباد نسبت به سایر اکوتیپ ها معرفی و پیشنهاد می گردد.یافته‌ها: نتایج بدست آمده نشان می دهد که بین مقادیر درصد اسانس اکوتیپ‌های مختلف مورد بررسی گونه دارویی مریم نخودی در سطح یک درصد اختلاف معنی‌داری وجود دارد. همچنین بین مقادیر ترکیبات شناسایی شده موجود در اسانس این گونه در رویشگاه های مختلف (اکوتیپ‌های متفاوت) اختلاف معنی‌داری در سطح یک درصد وجود دارد. ترکیبات اصلی اسانس اکوتیپ های مورد بررسی عبارتنداز: β-pinene، bicyclogermacrene، carvacrol، β-caryophyllene ،farnesene-cis-b ،germacren-D

Title: مطالعه شاخص‌ها و خصوصیات جوانه زنی بذر و استقرار دانه رست گیاه دارویی مورد (Myrtus communis L)

http://yafte.lums.ac.ir/article-1-755-fa.html

مقدمه: به دلیل اهمیت گیاه مورد در صنایع دارویی و همچنین کمک به احیای سریع‌تر این گیاه در طبیعت، کشت و کار آن از طریق بذر به عنوان روشی اقتصادی و کاربردی توصیه می‌گردد. با توجه به اینکه جوانه‌زنی بذر این گیاه مشکل می‌باشد و گسترش سطح زیر کشت و تولید بیشتر این گیاه را با مشکل مواجه می‌کند، از این رو در این پژوهش به بررسی عوامل ممانعت کننده جوانه زنی بذر این گیاه پرداخته شده است. بحث و نتیجه‌گیری: در این بررسی جداسازی بذرهای پوک و خراش دهی مکانیکی موجب حصول حداکثری جوانه زنی بذر و استقرار مطلوب گیاهچه‌ها شد. یافته‌ها: بررسی بذرهای سالم نشان داد که بیش از 76 درصدخواب آن ناشی از خواب فیزیکی و بقیه آن ممکن است مربوط به جنین و از نوع فیزیولوژیکی باشد. با وجودی که میزان جوانه زنی بذر در هر دو نوع خراش دهی تفاوت معنی داری نداشتند اما در مجموع استقرار گیاهچه های حاصل از تیمار خراش مکانیکی در مقایسه با خراش‌دهی شیمیایی بسیار بالاتر بود (88% در مقابل 55%) و خراش شیمایی اثرات مضری بر گیاهچه حاصله (خصوصاً بخش ریشه و بنیه آن) داشت. مواد و روش‌ها: برای رفع خواب فیزیکی (سخت پوستی) از تیمارهای خراش دهی مکانیکی (کاغذ سنباده) و شیمیایی (قرار دادن بذور در محلول اسید سولفوریک 98% به مدت 2 و4 دقیقه و سپس سه بار شستشو با آب مقطر)و برای رفع خواب فیزیولوژیک، یک و دو ماه سرمادهی اعمال گردید. این آزمایشها در قالب طرح کاملاً تصادفی با سه تکرار اجرا شد.
